# 3D cell sheet structure augments mesenchymal stem cell cytokine production

**DOI:** 10.1038/s41598-021-87571-7

**Published:** 2021-04-14

**Authors:** Sophia Bou-Ghannam, Kyungsook Kim, David W. Grainger, Teruo Okano

**Affiliations:** 1 Cell Sheet Tissue Engineering Center (CSTEC), Department of Pharmaceutics and Pharmaceutical Chemistry, Health Sciences, University of Utah, 30 South 2000 East, Salt Lake City, UT 84112 USA; 2 Department of Biomedical Engineering, University of Utah, 36 S Wasatch Dr, Salt Lake City, UT 84112 USA; 3 Institute of Advanced Biomedical Engineering and Science, Tokyo Women’s Medical University, 8-1 Kawada-cho, Shinjuku-ku, Tokyo, 162-8666 Japan

**Keywords:** Biomedical engineering, Regenerative medicine, Stem-cell biotechnology, Tissue engineering

## Abstract

Mesenchymal stem cells (MSCs) secrete paracrine factors that play crucial roles during tissue regeneration. An increasing body of evidence suggests that this paracrine function is enhanced by MSC cultivation in three-dimensional (3D) tissue-like microenvironments. Toward this end, this study explored scaffold-free cell sheet technology as a new 3D platform. MSCs cultivated on temperature-responsive culture dishes to a confluent 2D monolayer were harvested by temperature reduction from 37 to 20 °C that induces a surface wettability transition from hydrophobic to hydrophilic. Release of culture-adherent tension induced spontaneous cell sheet contraction, reducing the diameter 2.4-fold, and increasing the thickness 8.0-fold to render a 3D tissue-like construct with a 36% increase in tissue volume. This 2D-to-3D transition reorganized MSC actin cytoskeleton from aligned to multidirectional, corresponding to a cell morphological change from elongated in 2D monolayers to rounded in 3D cell sheets. 3D culture increased MSC gene expression of cell interaction proteins, β-catenin, integrin β1, and connexin 43, and of pro-tissue regenerative cytokines, vascular endothelial growth factor (VEGF), hepatocyte growth factor (HGF), and interleukin-10 (IL-10), and increased VEGF secretion per MSC 2.1-fold relative to 2D cultures. Together, these findings demonstrate that MSC therapeutic potency can be enhanced by 3D cell sheet tissue structure.

## Introduction

With the expansion of tissue engineering and regenerative medicine, mesenchymal stem cells (MSCs) have gained traction as effective candidates for repair and regeneration of injured tissues^1^. A unique characteristic of MSCs is their ability to secrete a wide range of bioactive molecules, including growth factors and cytokines, that can influence nearby cells via paracrine signaling to facilitate various biological processes desirable for tissue regeneration, such as angiogenesis, immune modulation, injured cell repair rather than death and scarring, cell proliferation, and cell recruitment and differentiation^2–5^. A growing body of clinical evidence attributes the therapeutic role of MSCs to their paracrine actions^6–8^. For example, clinical trials to treat ischemic heart failure have demonstrated that MSC injection into the border zone between infarcted and viable cardiac tissue resulted in potent antifibrotic effects, including augmentation of viable and perfused tissue, despite no injected MSC engraftment or differentiation; cardiac improvement is instead attributed to endogenous regeneration mechanisms stimulated by MSC-secreted bioactive factors^9^. Despite some clinical benefits, MSC engraftment following direct injection is generally low and transient in nature^10–12^. Without high transplantation efficacy of potent, clinically meaningful cell numbers, continuous paracrine factor delivery is not possible, thereby blunting the potential efficacy of MSC therapies^13^.

MSC cultivation with three-dimensional (3D) culture systems, predominantly scaffolds, hydrogels, and spheroids, is familiar for tissue engineering applications that overcome engraftment limitations of cell injections. Importantly, these systems have also been shown to enhance MSC paracrine activity through both morphological and molecular changes related to cell interactions compared to conventional two-dimensional (2D) adherent culture^14–16^. Tissue culture-treated 2D culture surfaces impose high basal adhesion and forced cell polarity; 3D culture systems diminish forced polarity and excessive basal spreading, allowing 3D cells to adopt a more physiologic morphology favorable for MSC paracrine function^17,18^. Furthermore, a well-accepted correlation exists between the 3D upregulation of cell–cell and cell–matrix interactions and increased MSC paracrine benefit compared to 2D monolayer culture. For instance, Qazi et al. demonstrated that MSC-seeded porous scaffolds increased in vitro MSC cytokine secretion and enhanced paracrine-mediated myoblast migration and proliferation compared to both 2D plastic-cultured MSCs and hydrogel encapsulated MSCs. This study attributed 3D cell–cell interactions to the enhancement of MSC paracrine effects^19^. Similar paracrine effects have been reported for MSC spheroids and specifically implicate intercellular adhesion proteins^20^ and gap junction proteins^21^ in MSC functional augmentation.

For engineering a MSC paracrine boost, however, current 3D culture systems limit cell interactions, interrupted by biomaterial barriers in scaffolds and hydrogels. Similarly, cells cultured in 3D hanging drop or pellet systems aggregate into spheroids primarily via cell–cell interactions, lacking cell–matrix interactions characteristic of native tissue structures and important to MSC paracrine capacity^22,23^. By limiting one or more of these important types of endogenous cellular interactions, present 3D culture systems inherently hinder innate MSC capabilities to boost intrinsic paracrine functions and signaling crucial for therapeutic goals.

Cell sheet tissue engineering, a method for generating 3D tissues, utilizes commercial temperature-responsive polymer-grafted cell culture dishes (TRCD)^24,25^ on which cells can be cultured using conventional 2D adherent culture practices. As the cells grow across the TRCD toward confluence, they deposit extracellular matrix (ECM) and form adhesion interactions and junctions with the ECM and with neighboring cells. At confluence, cells cultured on TRCD can be detached from the culture surface by aqueous temperature reduction from 37 to 20 °C, prompting a surface property change from hydrophobic to hydrophilic that releases adherent cells from the surface as a contiguous sheet, bypassing conventional enzymatic cell harvesting methods. Uniquely, the recovered cell sheet is characterized by retention of deposited ECM, cell adhesive proteins, and cell–cell and cell–matrix interactions generated during confluent culture^26–30^. Previous reports have shown that cells detached from TRCDs by temperature-reduction experience a morphological change due to reorganization of their actin cytoskeletal structures^31^. However, the impact of this cell sheet 3D transition on cell cytokine secretory function has not yet been studied.

Given preservation of culture-derived intercellular interactions, ECM, and cell–ECM interactions within a 3D tissue-like microenvironment^26,32,33^, we expected cell sheet technology to provide a new platform to boost MSC paracrine capacity. Specifically, the unique TRCD culture method induces a spontaneous 3D cell sheet transition following cell sheet detachment that increases both cell–cell and cell–matrix interactions relative to the 2D counterpart. Therefore, we evaluated this dynamic 3D transition that MSCs experience following cell sheet detachment and sheet spontaneous contraction from 2D adherent monolayer culture for cytoskeletal remodeling, morphological changes, and cell interaction variations, notably linked to MSC paracrine capacity.

## Results

### Cell sheet contraction into a 3D structure influences individual cell morphology

**Figure 1:**
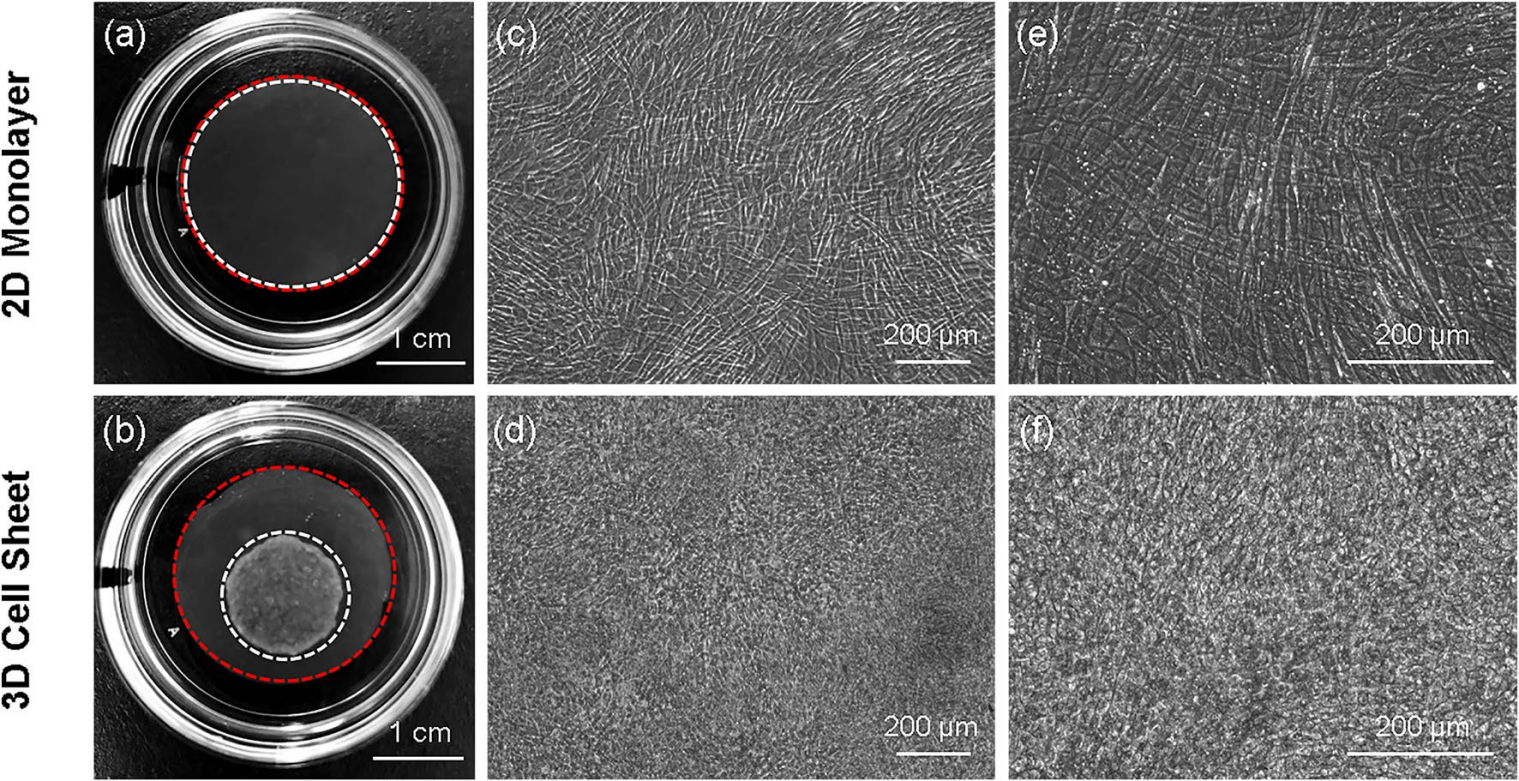
Microscopic cell morphology influences macroscopic tissue structure. Macroscopic image and microscopic cell morphology of hUC-MSC 2D monolayers seeded onto an insert membrane, and contracted 3D cell sheets following temperature-detachment and placement on an insert membrane. In both groups, hUC-MSCs were seeded at 41,580 cells/cm^2^ initial cell densities. Macroscopic images of a (**a**) 2D monolayer (white dashed circle) on an insert membrane (red dashed circle, 24 mm diameter) and a (**b**) 3D cell sheet (white dashed circle) on an insert membrane, placed in the center of tissue-culture plastic dishes (35-mm diameter) for imaging. Morphology of hUC-MSCs in a 2D monolayer at (**c**) × 10 and (**e**) × 20 magnification, and in a 3D cell sheet at (**d**) × 10 and (**f**) × 20 magnification, observed using phase-contrast microscopy. Scale bars = 1 cm in (**a**) and (**b**). Scale bars = 200 μm in (**c**) through (**f**).

Human umbilical cord mesenchymal stem cells (hUC-MSCs) are seeded onto a TRCD and grown to confluence under conventional adherent culture conditions, rendering a 2D monolayer. To generate and release a cell sheet, the 2D monolayer undergoes a 2D-to-3D transition: temperature reduction from 37 to 20 °C changes the TRCD surface from hydrophobic to hydrophilic, releasing the 2D monolayer from adherent culture tension and prompting contraction into a 3D cell sheet (Fig. 1b). The 2D monolayer before contraction was reproduced on a cell culture-insert membrane with identical culture conditions as the 3D cell sheet group. Immediately prior to cell sheet detachment/contraction, insert membrane-cultured monolayers were taken as 2D monolayer (Fig. 1a) controls. In parallel with the gross change in cell sheet macroscopic structure following detachment contraction, the cell morphology undergoes a transition from 2D aligned adherent cell shape (Fig. 1c,e) in 2D monolayers to 3D unaligned rounded cell shape (Fig. 1d,f) in 3D cell sheets. This result suggests that cell sheet contraction into a 3D structure post-release alters cell shape.

### Cell sheet contraction from an adherent monolayer yields a 3D tissue-like structure

**Figure 2:**
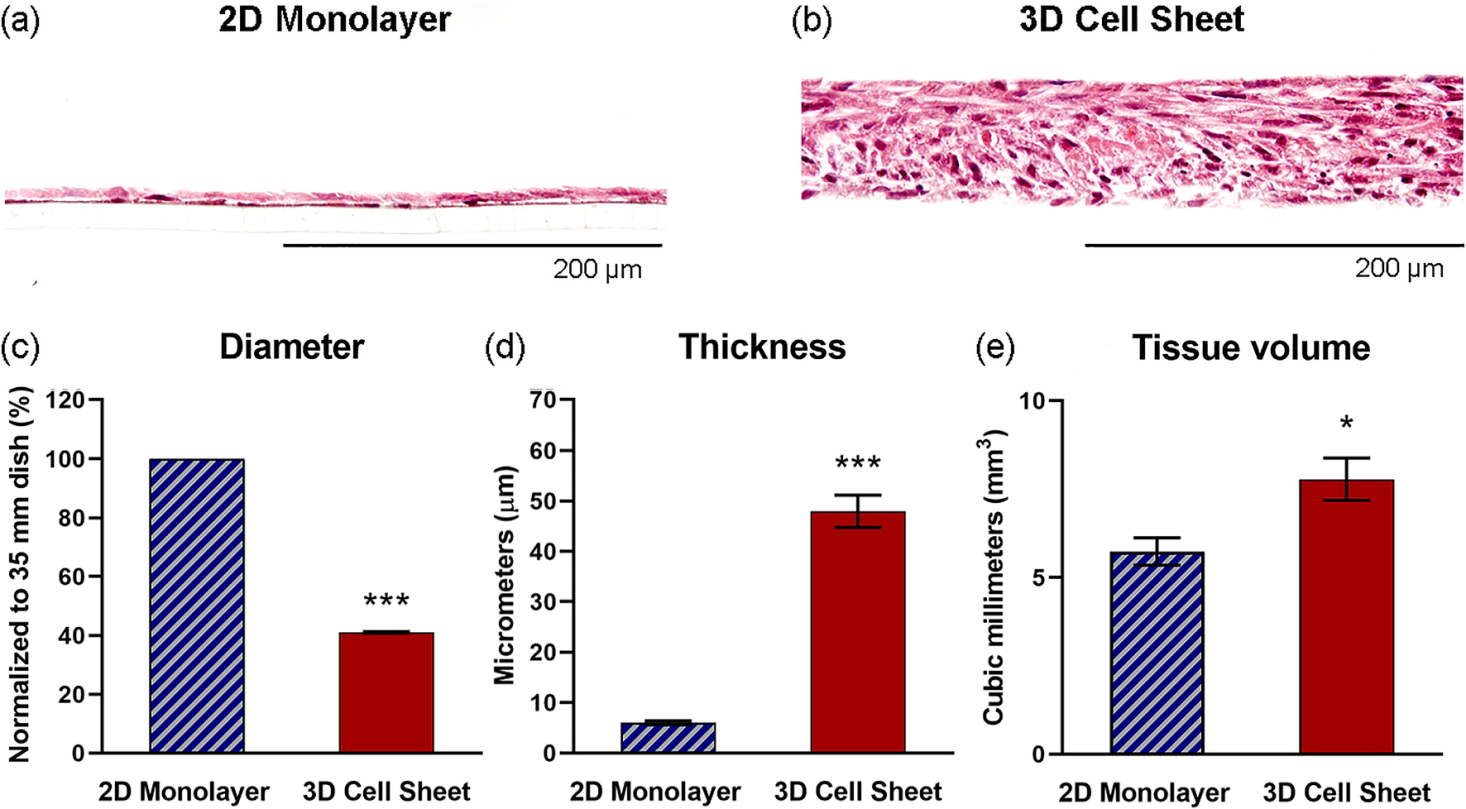
Spontaneous cell sheet contraction contributes a 3D tissue-like structure. Cross-sectional visualization of (**a**) 2D monolayer and (**b**) 3D cell sheet tissue structure with H&E stain. Quantified comparison of 2D monolayer and 3D cell sheet (**c**) diameter, (**d**) thickness, and (**e**) volume. Scale bars = 200 μm. Values are means ± SE (n = 10 (diameter), n = 3 (thickness): *p < 0.05, ***p < 0.001).

Hematoxylin and eosin (H&E) staining of hUC-MSC monolayer and sheet cross-sections shows that the 2D monolayers are, indeed, only single-nuclei thick, while the 3D cell sheets are multi-nuclei thick structures (Fig. 2a,b). Contracted hUC-MSC sheet 3D structure is contributed by a 2.4-fold reduction in sheet diameter (Fig. 2c) (*p* = 6.0 × 10^–18^) and an 8.0-fold increase in sheet thickness (Fig. 2d) (*p* = 4.4 × 10^–7^), representing a 36% increase in tissue volume (Fig. 2e) (*p* = 0.023), compared to the hUC-MSC 2D monolayer structure.

### hUC-MSC actin structure (cytoskeleton) changes in response to cell sheet contraction

**Figure 3:**
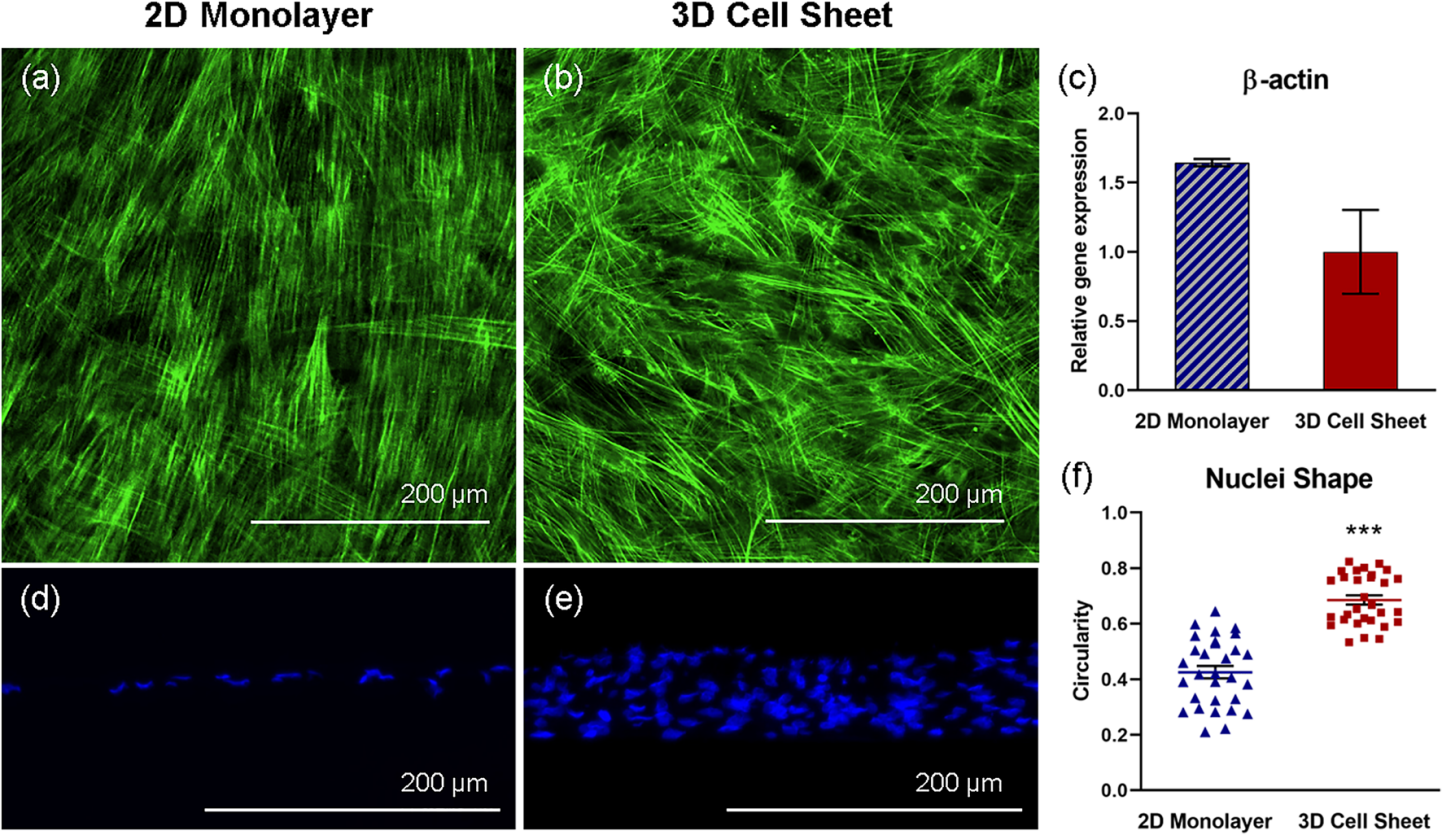
hUC-MSC actin structure changes in response to cell sheet contraction. Representative immunofluorescent images of hUC-MSC cytoskeleton (F-actin, green), imaged top-down, within (**a**) adherent 2D monolayers and (**b**) detached contracted 3D cell sheets. Cytoskeletal remodeling in response to cell sheet contraction is demonstrated by non-significant differences in (**c**) β-actin gene expression in hUC-MSCs in 2D monolayers and 3D cell sheets. hUC-MSC nuclei (DAPI, blue), imaged in cross-section, are elongated within the aligned cytoskeletal structure in (**d**) 2D monolayers and are significantly more rounded within the 3D cytoskeletal organization in (**e**) 3D cell sheets, evidenced by (**f**) nuclei circularity quantification. Scale bars = 200 μm. Values are means ± SE (n = 30: ***p < 0.001).

hUC-MSC 2D monolayer and 3D cell sheet cytoskeletal arrangement was observed with phalloidin (F-actin) fluorescent staining. Imaged from the top-down, hUC-MSCs in 2D monolayers exhibit unidirectional and elongated cytoskeletal structures, aligned in the direction of cell spreading (Fig. 3a). Conversely, hUC-MSCs that undergo cell sheet contraction present a more 3D cytoskeletal structure with random, multidirectional alignment (Fig. 3b). Although no significant differences in β-actin gene expression per hUC-MSC in each group were evident, hUC-MSCs in 2D monolayers showed greater average β-actin gene expression compared to hUC-MSCs in 3D cell sheets (Fig. 3c). Actin structure and gene expression differences indicate that hUC-MSC cytoskeleton remodeled toward a 3D arrangement in response to cell sheet contraction from the 2D adherent monolayer.

### hUC-MSC nuclear shape changes in response to cell sheet contraction

DAPI-visualized nuclei in 2D hUC-MSC monolayers appeared more elongated than nuclei in 3D hUC-MSC sheets (Fig. 3d,e). To quantify this finding, nuclei circularity was measured, where a value of 1.0 indicates a perfect circle, and values approaching 0.0 indicate an increasingly elongated shape. The average circularity of nuclei in 3D hUC-MSC sheets (0.69 ± 0.092) was closer to 1.0 than nuclei in 2D hUC-MSC monolayers (0.43 ± 0.12), representing a significant difference in nuclei circularity due to cell sheet contraction (Fig. 3f) (*p* = 2.1 × 10^–9^). Consistent with cytoskeleton remodeling data, hUC-MSC nuclei reconfigured to more circular shapes in 3D cell sheets relative to 2D monolayers.

### Enhanced pro-regenerative cytokine gene expression is related to 3D cell sheet tissue-like structure

**Figure 4:**
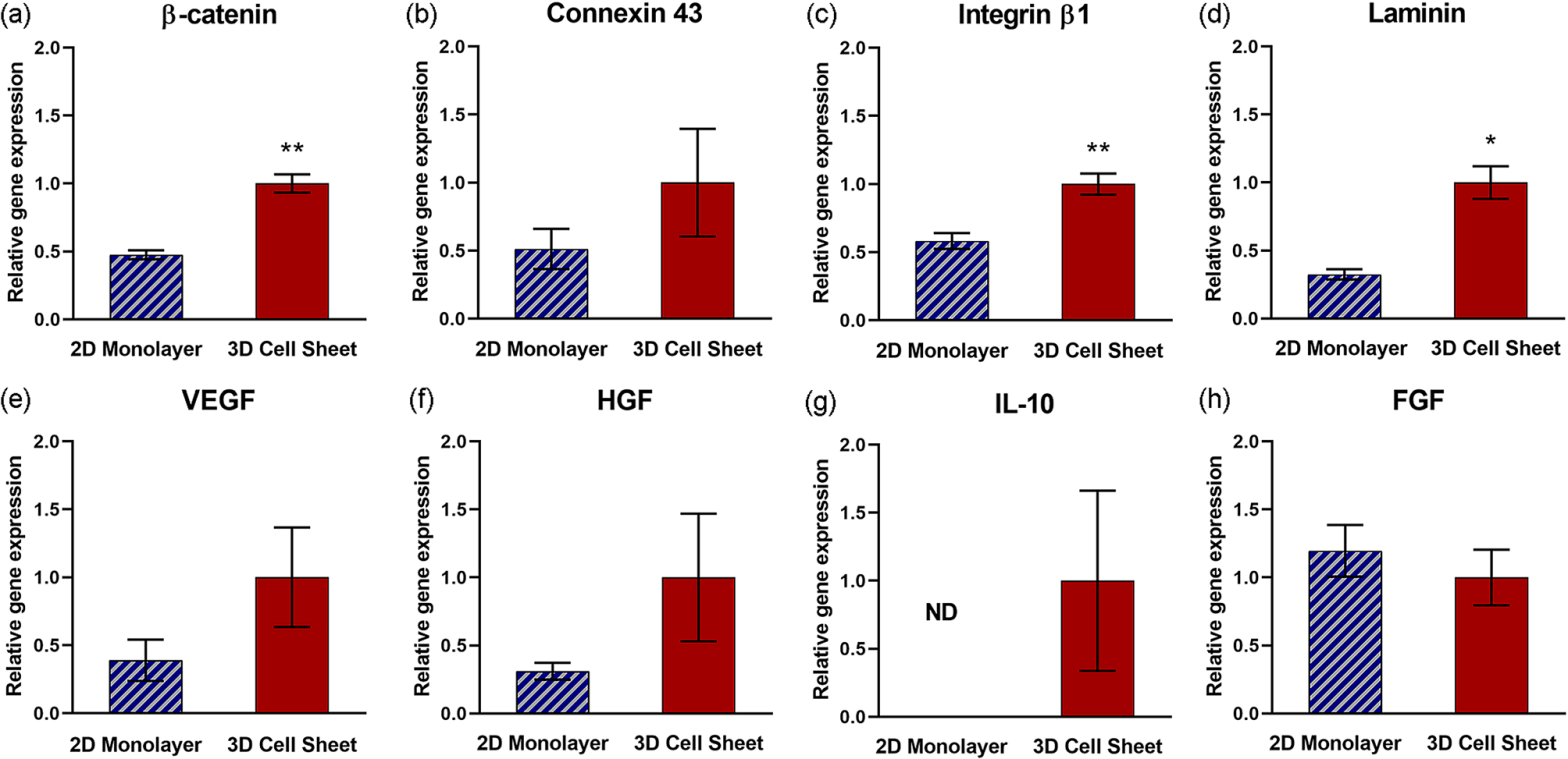
Enhanced pro-regenerative cytokine gene expression related to 3D cell sheet tissue-like structure. Quantitative gene expression of proteins related to tissue-like interactions, including (**a**) β-catenin (cell–cell interaction), (**b**) connexin 43 (gap junction), (**c**) integrin β1 (cell–ECM interaction), and (**d**) laminin (ECM), were increased per hUC-MSC in 3D cell sheets relative to 2D monolayers. Quantitative gene expression of pro-regenerative cytokines (**e**) VEGF, (**f**) HGF, and (**g**) IL-10 also increased per hUC-MSC in 3D cell sheets relative to 2D monolayers, with undetectable levels of IL-10 expression in hUC-MSCs in 2D monolayers. (**h**) FGF gene expression was similar in MSC 2D monolayer and 3D MSC sheets. Values are means ± SE (n = 3: *p < 0.05, **p < 0.01). *ND *not determined.

Cell interaction-protein gene expression appears to be upregulated in the 3D, tissue-like environment of contracted cell sheets. β-catenin, an intracellular portion of the adherens junction protein complex that binds extracellular cadherin to mediate cell adhesion to neighboring cells^34^, and integrin β1, a protein motif that extracellularly binds ECM ligands^35^, are both significantly upregulated in 3D cell sheets relative to 2D monolayers (Fig. 4a,c, respectively) (*p* = 0.0043 and *p* = 0.0051, respectively). Concomitantly, gene expression for cell-adhering ECM glycoprotein, laminin, is significantly upregulated in 3D cell sheets (Fig. 4d) (*p* = 0.036). Gene expression for connexin 43, a gap junction protein that spans the cell membranes of neighboring cells and allows direct intracellular cytoplasmic molecular signaling exchange, is increased on average per hUC-MSC in 3D cell sheets relative to 2D monolayers (Fig. 4b). Vascular endothelial growth factor (VEGF) and hepatocyte growth factor (HGF) gene expression per hUC-MSC are both upregulated on average in 3D cell sheets relative to 2D monolayers (Fig. 4e,f, respectively), although not significantly. Gene expression of interleukin-10 (IL-10) was undetectable in 2D monolayers but was measurable per hUC-MSC in 3D cell sheets (Fig. 4g). Fibroblast growth factor (FGF) gene expression was slightly increased on average per hUC-MSCs in 2D monolayers relative to 3D cell sheets (Fig. 4h). These data indicate that genes related to a tissue-like microenvironment are upregulated in 3D cell sheets compared to 2D monolayers, improving the pro-regenerative cytokine secretory capacity in 3D cell sheet hUC-MSCs.

### Cell sheet contraction increases real cytokine production by hUC-MSCs

**Figure 5:**
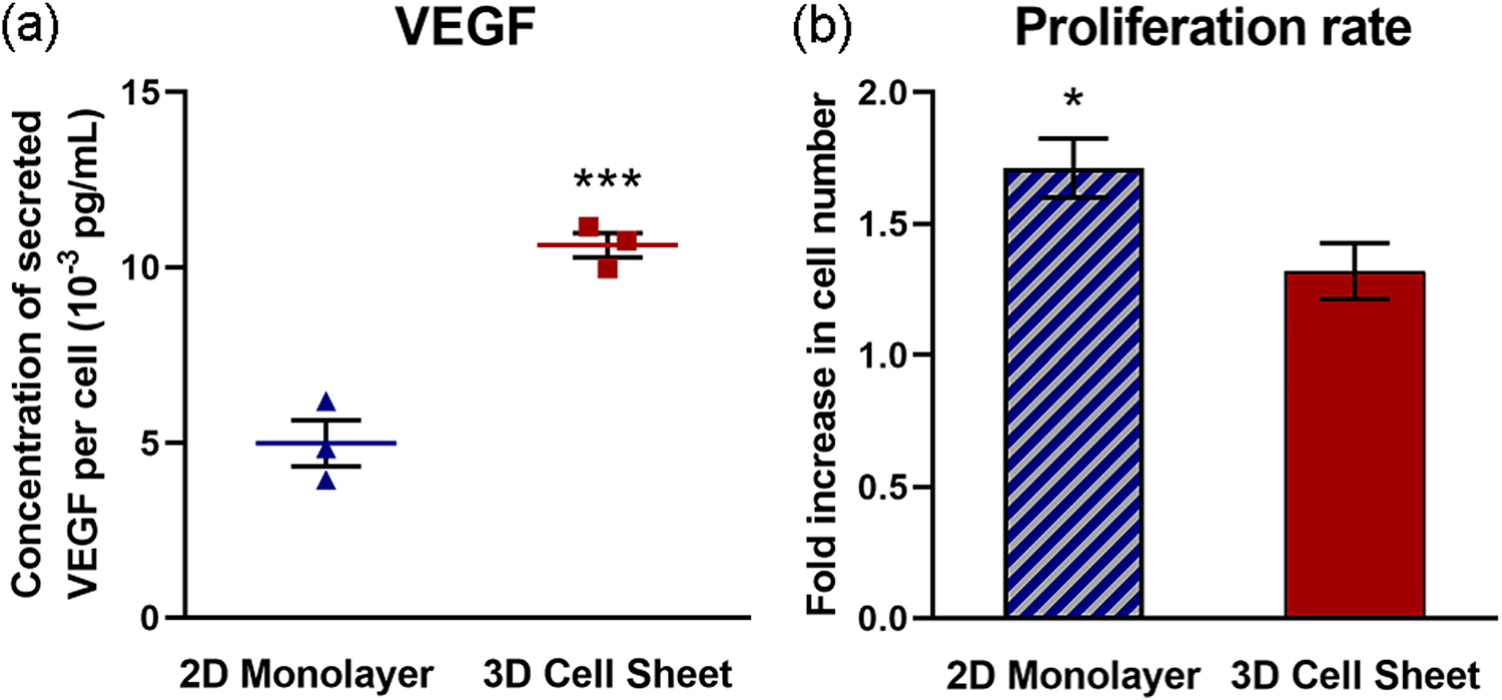
Cell sheet contraction increases actual cytokine production by hUC-MSCs. Human VEGF secretion was increased (**a**) 2.1-fold per hUC-MSC in 3D cell sheets compared to 2D monolayers cultured for 24 h. (**b**) The hUC-MSC proliferation rate during this time course was significantly higher in 2D monolayers compared to 3D cell sheets. Values are means ± SE (n = 3: *p < 0.05 and ***p < 0.001).

Human VEGF secretion per hUC-MSC was increased 2.1-fold in 3D cell sheets compared to 2D monolayers, measured over a 24-hour (h) culture span (Fig. 5a). To account for differences in cell numbers due to cell proliferation over 24 h, secreted VEGF was normalized to average cell number per group at 24 h. Despite a significantly higher cell proliferation rate in 2D monolayers (1.7-fold increased ± 0.20, *p* = 0.013) compared to 3D cell sheets (1.3-fold increased ± 0.18) over the 24-h culture span (Fig. 5b), 3D cell sheet hUC-MSCs secreted twice as much VEGF per cell on average.

## Discussion

Clinical MSC applications exploit the unique paracrine signaling function of MSCs^36–38^, where the therapeutic potency is dependent upon MSC cytokine secretory capacity as single cell-suspension formulations. Toward improving their clinical significance, we assert that engineering MSCs as tissues functionally enhances individual MSCs beyond their dissociated single cell potency. Specifically, MSC cytokine production is clearly stimulated using highly functional MSC-dense tissue-like constructs. This tissue effect was clearly demonstrated in a previous study that compared single MSCs to 2D MSC monolayers^33^. Single MSCs that had been enzyme-dissociated to cleave cell interactions between neighboring cells, as well as degrade ECM proteins and cleave associated cell-ECM binding proteins, were compared to 2D MSC adherent monolayers that preserved confluent cell–cell and cell–matrix interactions. Paracrine factors VEGF, HGF, and IL-10 were significantly upregulated in 2D MSC monolayer cultures relative to single dissociated MSCs^33^. This upregulation was clearly attributed to maintenance of a tissue-like microenvironment^33^. In the present study, we further explored the tissue effect on clinically important MSC cytokine production potency, pushing the tissue model one step further by contracting 2D monolayers into 3D cell sheets. The 3D tissue-like microenvironment supports physical (cell shape and spatial arrangement) and chemical (cell interaction and binding protein expression) effects that augment MSC potency beyond 2D monolayer and single cell formulations.

Spontaneous cell sheet contraction creates a 3D microenvironment by contracting the monolayer diameter 40% and increasing the thickness 8.0-fold, representing a 36% increase in tissue volume of the contracted cell sheet compared to the 2D monolayer (Fig. 2c–e). While the exact mechanism underlying this volume increase is unclear, cell attachment strength is likely implicated. During 2D adherent culture on tissue culture plastic, the cell–material interaction imposes a high basal adhesive force that compacts the cell cytoskeleton and promotes a tight-packed arrangement of cells and deposited ECM as the cells grow to confluence. The actin cytoskeleton uniformly aligns and compacts in the direction of cell spreading (Fig. 3a). Furthermore, cell attachment and spreading may be attributed to water efflux, resulting in reduced cell volume^39,40^. Once the cell–material interface is disrupted by temperature-responsive release from the material surface, cytoskeletal compaction is released and reorganizes to confer a 3D cell shape, with multidirectional actin arrangement (Fig. 3b). Changing MSC β-actin expression further evidences cytoskeletal rearrangement (Fig. 3c). β-Actin gene expression is relatively higher in 2D monolayer cells compared to those in the 3D cell sheet; this could be due to β-actin’s relationship to cytoskeletal tension, which would be much higher under plastic adherent culture than in tissue culture^41^.

Monolayer adherent culture restricts cell adhesion to 2D because of high basal adhesion, limiting cell–cell contacts to the perimeter of adjacent cells. In contrast, cells in 3D culture can make cell interactions in all directions, between the encompassing matrix and between neighboring cells^42^. For this reason, 3D culture provides higher abundance cell interactions relative to 2D conditions^43^. Consistently, our study demonstrated that MSCs in 3D cell sheets also increase cell interactions (Fig. 4a–c). Intracellular catenin, directly binding cytoskeletal F-actin, forms a transmembrane complex with extracellular cadherin to connect adjacent cells^34^. β-Catenin gene expression was significantly upregulated in 3D cell sheet MSCs relative to 2D monolayer MSCs, likely due to increased cell–cell interactions as well as in response to contraction-imposed change in actin cytoskeletal structure. Connexin 43, a major MSC gap junction protein^44^, is similarly upregulated upon increased cell–cell contact in 3D cell sheets. Furthermore, integrin β1, an extracellular adhesion protein connecting cells and ECM^45^, is upregulated in 3D cell sheet MSCs. This observed integrin β1 gene expression increase is consistent with cytoskeletal remodeling as well as an increase in laminin gene expression (Fig. 4d), suggesting that cytoskeleton-bound integrin β1 binds ECM component, laminin, to facilitate greater cell–ECM adhesion in 3D cell sheets relative to 2D monolayers.

MSC paracrine function is arguably one of their most clinically beneficial attributes^46,47^, mediated by MSC secretion of myriad pro-regenerative cytokines and subsequent paracrine activity in host tissue. VEGF, HGF, FGF, and IL-10 directly regulate tissue repair and regeneration, with specific implications in vascularization^48^, fibrosis mitigation^49^, cell regeneration^50^, and inflammation mediation^51^, respectively. MSC production of major therapeutic cytokines involved broadly across tissue regeneration was assessed: 3D cell sheets increased pro-tissue regenerative VEGF, HGF, and IL-10 gene expression (Fig. 4e–g), while IL-10 was undetectable in MSCs in 2D monolayers. VEGF production doubled per MSC in contracted 3D cell sheets relative to 2D monolayers (Fig. 5a), despite significantly higher MSC proliferation rates in 2D monolayers (Fig. 5b). This difference in proliferative activity is to be expected, as it is widely recognized that 2D adherent culture generally promotes stromal cell proliferation rates faster than 3D culture conditions due to excessive basal adherence and cell spreading^39,52^. Normalizing for cell number and proliferation, we attribute greater cytokine-production potency to a 3D tissue effect: in part due to structural changes in cell morphology and cytoskeletal tension, and partly due to chemical cell interactions that are both deficient in 2D culture and absent in single cell suspension^19,53,54^. Particularly, β-catenin plays a specific role in mediating adipose-derived MSC HGF secretion via enhanced cell–cell adhesion^55^. Gap junction proteins that allow direct molecular signal exchange across lipid membranes of neighboring cells have been similarly identified for their key role in boosting individual MSC VEGF secretion, promoting angiogenesis^56^. Also, tissue-like cell–cell and cell–ECM interactions within 3D MSC culture systems significantly improved immune mitigation in an inflammatory arthritis model, due to notable upregulation of MSC-secreted IL-10^57^. FGF is strongly related to MSC proliferative activity^58^; based on this published evidence combined with FGF expression and MSC proliferative data shown in Figs. 4 and 5 contrasting 2D and 3D MSC properties, 2D adherence mediated MSC proliferation would be expected to yield similar FGF gene expression in both 2D monolayer and 3D cell sheet MSCs (Fig. 4h).

**Figure 6:**
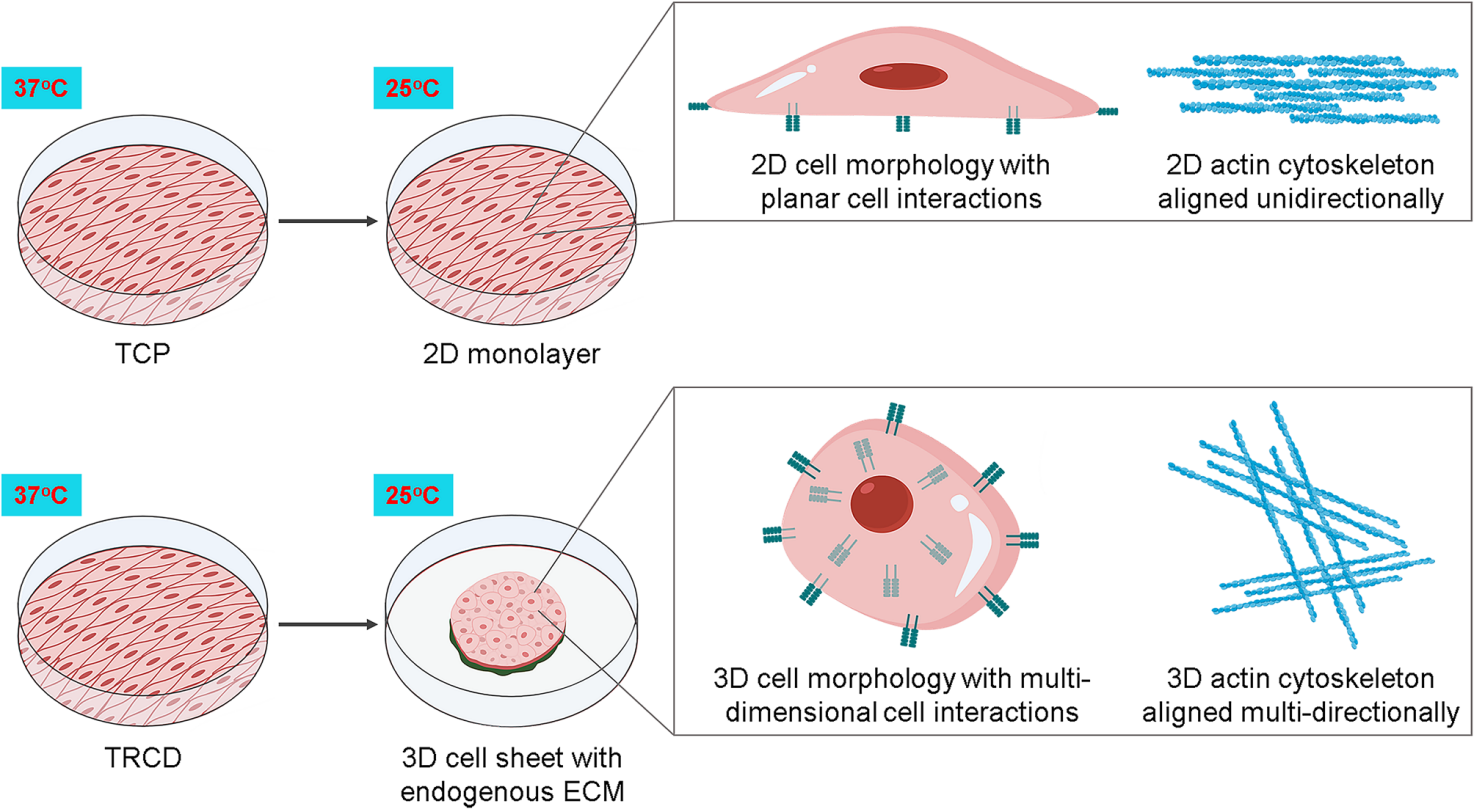
MSC sheet 3D structural and molecular transition. A schematic representation of TRCD-cultured MSC sheet temperature detachment and sheet contraction resulting in a tissue-like 3D system. 2D–3D transition prompts dynamic cell morphology and actin cytoskeleton changes with increased cell–cell interactions. These features of 3D MSC sheet culture and production augment MSC paracrine-relevant secretory capacity. Created with BioRender.com.

Taken together, our results highlight several key features of cell sheet technology that collectively augment MSC cytokine secretory function: (1) temperature-induced detachment and spontaneous MSC monolayer contraction produces a 3D construct by spontaneous structural and morphological 3D transitions, (2) this 3D transition increases cell–cell and cell–matrix interactions endogenously derived during cell sheet fabrication, and (3) this 3D tissue effect enhances MSC cytokine secretory capacity relative to 2D MSC culture conditions (Fig. 6). For these reasons, cell sheet technology represents a 3D culture platform that enhances MSC paracrine capacities attributed to improved MSC clinical utility.

## Conclusions

Spontaneous cell sheet contraction upon release from adherent 2D monolayer culture produces a 3D tissue-like microenvironment that facilitates a 3D MSC shape and cytoskeletal organization. Additionally, this 3D transition upregulates MSC cell–cell, cell–ECM, and gap junction interactions. 3D cell sheet culture increases MSC paracrine activity due to a tissue effect, characterized by cell-experienced structural and chemical changes. As a 3D MSC cultivation system, cell sheets are a promising new platform to boost MSC paracrine effects without exogenous biomaterials and without sacrificing crucial cell–matrix interactions. Collectively, these findings describe a 3D-engineered tissue with enhanced MSC paracrine-relevant secretory function relative to adherent monolayer MSC culture.

## Materials and methods

### hUC-MSC culture

Banked hUC-MSCs isolated from the subepithelial layer of human umbilical cord tissue (Jadi Cell LLC, FL, USA, IRB-35242) were initiated at 4,500 cells/cm^2^ and expanded in growth media containing Dulbecco’s Modified Eagle’s Medium (Life Technologies, CA, USA) supplemented with 10% fetal bovine serum (FBS) (Thermo Fisher Scientific, MA, USA), 1.0% penicillin streptomycin (Gibco, NY, USA), 1.0% Glutamax (Life Technologies), and 1.0% non-essential amino acids (Life Technologies), and incubated in a humidified environment (37 °C, 5.0% CO_2_). Media was changed after 24 h of initiating culture and every 2 days subsequently. hUC-MSCs were passaged upon reaching 85% confluence.

### hUC-MSC 2D monolayer and 3D cell sheet fabrication and morphological analysis

Passage 5 hUC-MSCs were passaged using 0.05% Trypsin–EDTA (Gibco) and the cell suspensions were counted using a hemocytometer. The resultant passage 6 hUC-MSCs were aliquoted in 20% FBS growth media supplemented with 50 μg/mL l-ascorbic acid 2-phosphate (Sigma-Aldrich, MO, USA). P6 hUC-MSCs were seeded at 41,580 cells/cm^2^ onto 1.0 μm-diameter pore, 6-well cell culture insert membranes (Falcon, NE, USA) and onto 35-mm diameter UpCell TRCDs (CellSeed, Tokyo, Japan) and cultured for 4 days in a humidified environment without exchanging media. At 4 days, hUC-MSCs cultured on 6-well cell culture insert membranes underwent fresh growth media supplemented with 10% FBS exchange, yielding 2D monolayer samples, and, simultaneously, hUC-MSCs cultured on TRCDs were moved to 20 °C and spontaneously detached within 30 minutes (min), generating 3D cell sheet samples. Immediately following detachment, 3D cell sheets were transferred onto pre-coated (FBS-coated overnight, then rinsed twice with PBS) 6-well cell culture insert membranes and allowed to adhere for 1 h in standard incubation conditions (37 °C, 5.0% CO_2_). After 1-h incubation, fresh growth media supplemented with 10% FBS was added into 6-well cell culture inserts. At this time (experimental time 0-h), cell morphologies of the 2D monolayer and 3D cell sheet samples on respective 6-well cell culture insert membranes were imaged using phase contrast microscopy (AX10 microscope, Carl Zeiss Microimaging, Göttingen, Germany). To measure 3D cell sheet diameter at time 0-h, macroscopic pictures of hUC-MSC sheets were captured top-down immediately after detachment (n = 10 cell sheets per group). The scale was normalized to the 35-mm diameter TRCD in each image and 10 linear measurements of sheet diameter per image were recorded using ImageJ (U. S. National Institutes of Health, MD, USA).

### hUC-MSC 2D monolayer and 3D cell sheet tissue structural analysis

2D monolayers and 3D cell sheets on respective 6-well cell culture insert membranes in growth media with 10% FBS were cultured for an additional 24 h (experimental time 24-h) after fabrication to be used for structural analysis. Samples at 24 h were fixed with 4.0% paraformaldehyde (PFA) (Thermo Scientific) for 30 min and paraffin embedded. Embedded samples were sectioned at 4.0 μm thickness and stained with Mayer’s Hematoxylin (Sigma-Aldrich) and Eosin (Thermo Scientific) to visualize the tissue dimensions in cross section. Stained tissue sections were dried overnight and imaged with a Bx41 widefield microscope (Olympus, Japan) using AmScope Software (AmScope, CA, USA). To calculate 2D monolayer and 3D cell sheet thicknesses, 5 H&E pictures were taken along the length of the monolayer or cell sheet (n = 3), and 5 linear measurements from the apical to basal plane of the monolayer or cell sheet were made per picture using AmScope Software (AmScope) and averaged per group. Tissue volume was calculated using 10 measurements of thickness and diameter per group. Percent change in tissue volume was calculated from the average volumes.

### Immunofluorescent staining

To analyze differences in the cytoskeletal orientation of hUC-MSCs as a function of cell sheet contraction into a 3D structure, 24-h 2D monolayers and 3D cell sheets were fixed on the insert membrane with 4.0% PFA for 30 min, permeabilized with 0.1% Triton X-100 (Sigma-Aldrich) in PBS for 15 min, then washed with 1 × PBS and incubated with AlexaFluor 488 phalloidin (1:100, F-actin probe, Life Technologies) at room temperature (RT), blocked from light exposure, for 30 min. The phalloidin immunostained cell sheets were visualized for actin structure using top-down confocal microscopy (Nikon A1 and NIS-Elements Advanced Research software, Nikon Instruments, Tokyo, Japan). To visualize nuclei shape, cross-section samples were stained with DAPI (Life Technologies). Briefly, cross-section samples were deparaffinized before permeabilization with 0.1% Triton X-100 (Sigma-Aldrich) in PBS for 15 min, then washed with 1 × PBS and incubated with DAPI solution (2 drops/mL) at RT for 5 min. DAPI samples were then washed with 1 × PBS and mounted with ProLong Diamond Anti-Fade Mountant (Invitrogen). DAPI-visualized nuclei were imaged with a Zeiss Axio widefield microscope and Zen software (Carl Zeiss Microimaging).

### Nuclei shape measurement

Nuclei circularity was quantified from DAPI-visualized nuclei in hUC-MSC 2D monolayer and 3D cell sheet cross-sectional images. Images were threshold and the “particle counter” function in ImageJ software was used to measure the circularity of particles with an aspect ratio between 0.0 and 1.0, with 0.0 corresponding to a completely elongated object and 1.0 corresponding to a perfect circle. Ten nuclei were measured per group (n = 3 sheets/group) and reported with mean and standard error (SE).

### Real-time quantitative polymerase chain reaction (PCR)

Total RNA was isolated from hUC-MSCs in 24-h 2D monolayer and 3D cell sheet samples (n = 3 sheets per group) in TRIzol (Ambion, Life Technologies, CA, USA) with the PureLink RNA Mini Kit (Invitrogen, Thermo Fisher Scientific) according to manufacturer instructions. Isolated RNA was quantified with a NanoDrop Spectrophotometer (Thermo Scientific) and all cDNA samples were prepared from 1.0 μg of RNA/sample using a high-capacity cDNA Reverse Transcription Kit (Life Technologies). Genes were quantified by real-time PCR using Applied Biosystems primers (glyceraldehyde 3-phosphate dehydrogenase [GAPDH, Hs99999905_m1] as a housekeeping gene, VEGF [Hs99999070], HGF [Hs00379140_m1], IL-10 [Hs00961622_m1], FGF2 [Hs00266645_m1], β-actin [Hs99999903_m1], β-catenin [Hs00355049_m1], integrin β1 [Hs01127536_m1], connexin 43 [Hs04259536_g1], and laminin [Hs00966585_m1]) and was performed on Applied Biosystems Step One Plus (Applied Biosystems, CA, USA). Relative gene expression was determined using the comparative threshold cycle (*C*_T_) change algorithm normalized to 24-h 3D cell sheets.

### Soluble VEGF secretion normalized to cell number and cell proliferation rate

Immediately following cell sheet detachment and re-plating onto insert membranes, 2D monolayer and 3D cell sheet media were exchanged for fresh growth media with 10% FBS and samples were cultured for 24 h (37 °C, 5.0% CO_2_). FBS media (10%) alone were cultured for 24 h as a control. The 24-h supernatants (n = 3 per group) were collected and aliquoted, then centrifuged at 1200 RPM for 5 min to pellet cellular debris and preserve soluble proteins in the supernatant. The concentration of soluble VEGF secreted per 2D monolayer and 3D cell sheet was quantified using a human VEGF Quantikine ELISA kit (R&D Systems, MN, USA) and normalized to cell numbers to determine VEGF secreted per cell. Briefly, samples were rinsed twice with PBS and trypsin–EDTA (0.05%, 2 mL) (Gibco) was added directly on the samples to be incubated at 37 °C first for 10 min in a humidified incubator, then for 15 min in a 37 °C water bath. Afterwards, trypsin was removed by centrifugation (1200 RPM, 5 min) and supernatant aspiration. Cell pellets were dispersed with 0.5 mL collagenase P (0.05%, Sigma Aldrich) and incubated for 10 min in a 37 °C water bath. At this point, a single cell suspension had been rendered. Cell suspensions were reconstituted to 1.0 mL with 10% FBS media and exact cell numbers were counted using a trypan blue exclusion assay (Cell Culture Tested Trypan Blue Solution, Sigma Aldrich). Proliferation rate was quantified as the fold change increase in cell number from 0 to 24 h.

### Statistical analysis

All statistical analysis was conducted on data sets of n ≥ 3 biological replicates, with quantitative values expressed as a mean ± SE. D’Agostino–Pearson omnibus K2 test was used to determine a normal distribution for each data set, and therefore a parametric analysis of significance was appropriate. A two-tailed, paired, Student’s *t* test was used to measure statistical significance using GraphPad Prism version 9.0.0 for Windows (GraphPad Software, San Diego, California USA, http://www.graphpad.com). Statistical significance was defined as *p < 0.05, **p < 0.01, and ***p < 0.001. No statistical significance was defined as *p* > 0.05.
